# Incidence of radiodermatitis and factors associated with its severity in women with breast cancer: a cohort study^☆^

**DOI:** 10.1016/j.abd.2023.01.004

**Published:** 2023-08-30

**Authors:** Loren Giagio Cavalcante, Rejane Aparecida Rodrigues Domingues, Batista de Oliveira Junior, Marco Antônio Rodrigues Fernandes, Eduardo Carvalho Pessoa, Luciana Patrícia Fernandes Abbade

**Affiliations:** a Department of Nursing, Faculdade de Medicina, Universidade Estadual Paulista, Botucatu, SP, Brazil; b Sector of Radiotherapy, Hospital das Clínicas de Botucatu, Botucatu, SP, Brazil; c Department of Infectology, Dermatology, Diagnostic Imaging and Radiotherapy, Faculdade de Medicina, Universidade Estadual Paulista, Botucatu, SP, Brazil; d Department of Gynecology and Obstetrics, Faculdade de Medicina, Universidade Estadual Paulista, Botucatu, SP, Brazil

**Keywords:** Breast neoplasm, Radiodermatitis, Radiotherapy, adjuvant, Risk factors

## Abstract

**Background:**

One of the main adverse reactions of adjuvant radiotherapy for breast cancer is radiodermatitis.

**Objective:**

To assess the incidence of radiodermatitis in women with breast cancer, identify factors associated with its severity and determine the time at which this event occurs.

**Methods:**

Prospective cohort study in 113 women with breast cancer who were evaluated before radiotherapy and at every fifth session until the end of treatment. Logistic regression and Cox proportional regression model were used for the assessment of risk factors; P values < 0.05 were considered significant.

**Results:**

The incidence rate of radiodermatitis was 98.2% and it was demonstrated that for each additional point of the Body Mass Index (BMI), the chance of occurrence of grades II to IV radiodermatitis increases by 14% (OR = 1.14 [95% CI 1.04–1.26]; p = 0.004) and statin use increases the risk of more severe skin lesions by four-fold (OR = 4.27 [95% CI 1.11–16.42]; p = 0.035). The exclusive use of hydrogel for skin hydration was an independent factor in delaying the onset of radiodermatitis (HR = 0.55 [95% CI 0.36–0.82]; p = 0.004).

**Study limitations:**

The main limitation of this study was its external validity. The identified factors should be considered for services and populations similar to those in this study.

**Conclusions:**

There was a high incidence of radiodermatitis and its severity was related to higher BMI, statin use; there was a protective effect of hydrogel use.

## Introduction

Breast cancer is the most common malignancy among women, excluding non-melanoma skin cancers. Radiotherapy plays an important role in locoregional and distant control of this neoplasm, increasing disease-free survival and overall survival rates by up to 20% in 20 years.^1^, ^2^ Therefore, radiotherapy is indicated for all patients submitted to conservative surgery and for those undergoing mastectomy.

One of the main adverse reactions of adjuvant radiotherapy in the breast is radiodermatitis. Approximately 85%‒90% of the patients develop some degree of this reaction, which can manifest itself with variable severity. Radiation promotes several skin changes, including increased pH, decreased hydration, increased cutaneous blood flow, and hyperpigmentation.^3^, ^4^, ^5^ Skin toxicities manifest as erythema, hyperpigmentation, desquamation, exulceration, ulceration, and necrosis.^6^, ^7^

The consequences of radiodermatitis are numerous and include decreased quality of life due to pain and discomfort, infection, decreased self-image, and radiotherapy interruption, impairing neoplasm control.^8^

Several factors have been associated with the cutaneous toxicity of radiotherapy, such as the three-dimensional breast shape and the treatment technique.^9^, ^10^ Comorbidities, concomitant chemotherapy, sun exposure, high-dose radiation, tumor location, and staging have been identified as related to radiodermatitis.^11^, ^12^, ^13^, ^14^

Iwakawa et al. demonstrated that the risk of skin reactions in the breast was highly dependent on the institution where the patient was treated due to different treatment techniques, surgical procedures, magnitudes of photon and electron energy, use of multi-blade collimators, immobilization devices, and wedge filters.^9^

Therefore, it is necessary to better understand the factors associated with the development of radiodermatitis in these patients, considering the different realities of radiotherapy services and the different populations served. In this context, this study aimed to evaluate the incidence of radiodermatitis in women with breast cancer, identify the factors associated with its severity and determine the time in which this adverse reaction occurs during radiotherapy.

## Methods

### Study type, location and participants

This was a prospective and analytical cohort study carried out in the radiotherapy sector of the Clinical Hospital of Botucatu Medical School – São Paulo State University “Júlio de Mesquita Filho. Inclusion criteria were women over 18 years of age, diagnosed with breast cancer, and an indication of radiotherapy with photon emission by the radiotherapist. This indication was for adjuvant radiotherapy treatment after an interval of 21 days post-chemotherapy, complete healing of the surgical wound, and free movement of the ipsilateral upper limb. Patients with skin changes in the breast prior to radiotherapy were excluded due to interference in the evaluation, as well as patients with limited mobility due to the difficulty in performing the study procedures.

The study was approved by the Research Ethics Committee of the University Hospital (CAAE: 62865916.2.0000.5411) and conducted in accordance with the recommendations of Resolution n. 466/12 (Declaration of Helsinki). The informed consent form was read and signed by the patients or their guardians before inclusion in the study.

### Study procedures

The participants were assessed at the time of inclusion in the study, before starting radiotherapy, and close to every five sessions (with intervals ranging from three to seven sessions) until the end of treatment.

In the first evaluation, demographic and clinical data, breast cancer staging (according to the American Joint Committee on Cancer – AJCC 8^th^ edition),^15^ radiotherapy regimen, and photographs of the breasts before treatment were collected. Subsequent evaluations included data on skin care, direct observation of the irradiated site, and photographs to assess the possible development of radiodermatitis. At the beginning and during radiotherapy, patients were instructed about treatment goals, care for the irradiated area, and adverse events. Because it is an observational study, there was no interference in the routine and prescriptions of the service.

### Outcomes

Primary outcome: incidence of radiodermatitis during standard and booster treatment, as well as degree of severity of the lesions.

Secondary outcomes: i) Factors associated with the development of radiodermatitis according to different classifications; ii) Time when radiodermatitis occurred during radiotherapy.

The diagnosis and classification of radiodermatitis in patients with breast cancer were performed according to criteria of the Radiation Therapy Oncology Group (RTOG)^16^: a) Grade I, mild erythema, epilation, and dry desquamation; b) Grade II, painful erythema, localized wet desquamation, and moderate edema; c) Grade III, confluent wet desquamation and significant edema; or d) Grade IV, ulceration, hemorrhage, and necrosis.

### Radiotherapy

Radiotherapy was performed using the Eclipse™ computerized treatment planning system (versions 11.0 and 13.0) from Varian Medical Systems. The two-dimensional radiotherapy technique with a linear accelerator, model Clinac® 2100C, by Varian, was used. The treatment was performed with X-ray beams (photons) of 6 MV with two or four tangent radiation fields. Four radiation fields were used, two with a wedge filter and two without the filter. All patients were treated with the SSD (Source Surface Distance) technique, in which the distance from the radiation source to the patient's skin is equal to the distance from the isocenter of the linear accelerator (source distance = 100.0 cm).

### Variables

The information on the variables was collected by a single researcher and was obtained from the patient's medical records and through an interview guided by a data collection form prepared by the researchers. Sociodemographic and clinical variables (age, ethnicity, smoking status, weight, height, body mass index, comorbidities, medications used) related to radiotherapy and breast cancer and variables related to skin care were collected.

### Statistical analysis

Categorical variables were presented as percentages and continuous variables as means and standard deviations when parametric and as medians and first and third quartiles when non-parametric. Due to the smaller number of patients with grades II to IV radiodermatitis, patients were divided into two groups for comparison: a) Group 1, patients with grade I radiodermatitis, and b) Group 2, patients with grades II to IV radiodermatitis. To compare clinical and demographic characteristics, the chi-square or Fisher exact test were used for categorical variables, the t-test for independent samples for continuous parametric variables, and the Kruskal-Wallis test for non-parametric ones. To assess risk factors for the development of higher degrees of radiodermatitis, a multivariate logistic regression analysis was performed, with variables with p < 0.1 being included in the model.

To assess the risk of developing radiodermatitis as a function of the duration of treatment, Cox proportional regression model was applied in the univariate and multivariate analyses, and for the latter, those with p < 0.1 were used in the univariate analysis. The Kaplan-Meier analysis was used to plot the survival curve for the significant variables in the Cox model.

All statistical analyses were performed using SPSS software, version 23.0 (IBM) and Epi Info™ (US Centers for Disease Control and Prevention). P-values < 0.05 were considered significant.

## Results

A total of 122 women with breast cancer were admitted to the radiotherapy service from February to September 2017. Nine patients were excluded: four due to treatment with electron emission, three due to skin changes secondary to the tumor, and two due to limited mobility. Therefore, 113 patients remained in the study.

The demographic, clinical, and radiotherapy characteristics of the 113 participants are shown in Table 1. The patients received standard radiotherapy, totaling 9 to 30 sessions, with a mean of 26 sessions (SD ± 2.8) and a mean dose of 5,727.4 (SD ± 4466.1) centigrays (cGy).

**Table 1:** Demographic, clinical, and therapeutic characteristics of 113 women with breast cancer submitted to photon radiation therapy.

Parameter	Value	Parameter	Value
Age (years)	57.1 ± 13.4	Tumor staging	
White women	104 (92.1)	0	02 (1.8)
Level of schooling (years of education)		I	30 (27.5)
≤ 4	38 (33.6)	II	44 (40.4)
5–8	27 (23.9)	III	30 (27.5)
9–11	22 (19.5)	IV	03 (2.7)
≥ 12	26 (23.0)	Previous chemotherapy	
Smoking	14 (12.4)	Not performed	24 (21.2)
Body Mass Index (kg/m²)	29.8 ± 5.7	DCT^a^	69 (61.1)
Comorbidities		Other regimens b	20 (17.7)
Diabetes mellitus	24 (21.2)	Therapy with tamoxifen	36 (31.8)
Arterial hypertension	66 (58.4)	Therapy with anastrozole	43 (38.1)
Heart	09 (8.0)	Performed surgery	
Dyslipidemia	13 (11.5)	Mastectomy	18 (15.9)
Hypothyroidism	15 (13.3)	Adenectomy	10 (8.8)
Drugs for continuous use		Setorectomy	81 (71.7)
Hypoglycemic agents	24 (21.2)	Radiation dose (cGy)	5.727.4 ± 4.466.1
Anti-hypertensive drugs	63 (55.7)	Dose variation (%)	6.8 ± 3.7
Analgesics	08 (7.1)	Radiated field (cm)	
Thyroid drugs	15 (13.3)	Depth	20.3 ± 2.9
Statins	14 (12.4)	Height	7.7 ± 1.4
Vitamins	25 (22.1)	Length	16.4 ± 1.8
Tumor histopathology		Radiated volume (cm^3^)	2.633.4 ± 869.4
Carcinoma *in situ*	06 (5.3)	Skin hydration	
Invasive carcinoma	99 (87.6)	Hydrogel only	50 (44.2)
Lobular carcinoma	05 (4.4)	Hydrogel with moisturizer	63 (55.8)
Special carcinoma	03 (2.6)	Mean oral hydration (L/day)	2.0 ± 0.6

Categorical variables were presented as absolute numbers and percentages (in parentheses) and continuous variables as mean and standard deviation.

a DCT ‒ Doxorubicin, Cyclophosphamide and Taxane (paclitaxel or docetaxel).

b Other regimens ‒ doxorubicin and cyclophosphamide; doxorubicin, cyclophosphamide and fluorouracil; doxorubicin, cyclophosphamide, taxane and carboplatin; docetaxel and carboplatin; cyclophosphamide, fluorouracil and methotrexate; gemcitabine and vinorelbine; cyclophosphamide and docetaxel.

Of the 113 patients, 68 (60.2%) also received booster treatment after completion of the standard radiotherapy, with a mean radiation dose of 1,202 (SD ± 323.2) cGy. The maximum dose on the skin ranged from 3% to 11% of the prescribed dose.

In addition to radiotherapy performed on the breast topography, 40 patients also received treatment with photon radiation on the ipsilateral supraclavicular fossa, due to the presence of lymph node metastases. It should be noted that because it is two-dimensional radiotherapy, there was no irradiation to the lymph node chain of the internal mammary gland and axilla.

### Primary outcome

**Figure 1:**
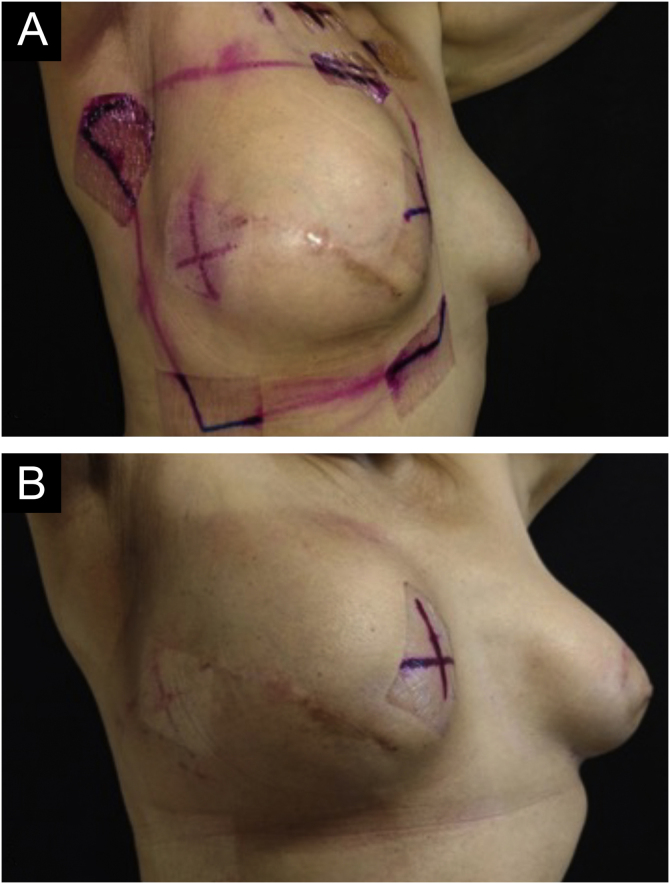
Images of the breast submitted to radiotherapy. (A) Before the first radiotherapy session. (B) After 28 sessions of radiotherapy with a total dose of 5271 cGy – grade I radiodermatitis is observed, characterized by mild erythema and hyperpigmentation.

**Figure 2:**
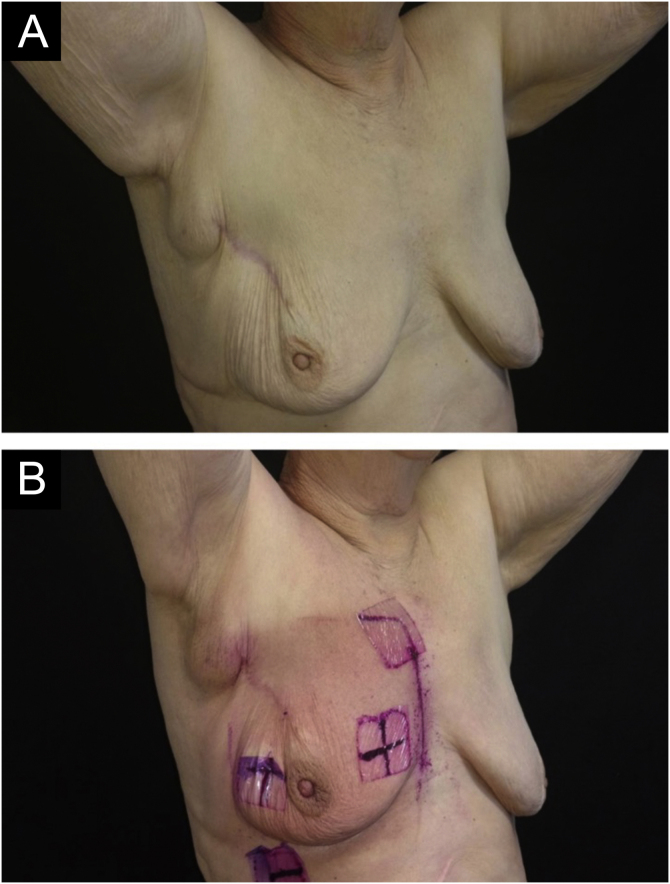
Images of the breast submitted to radiotherapy. (A) Before the first radiotherapy session. (B) After 28 radiotherapy sessions with a total dose of 5221 cGy – grade II radiodermatitis is observed, characterized by intense and painful erythema and moderate nipple edema.

**Figure 3:**
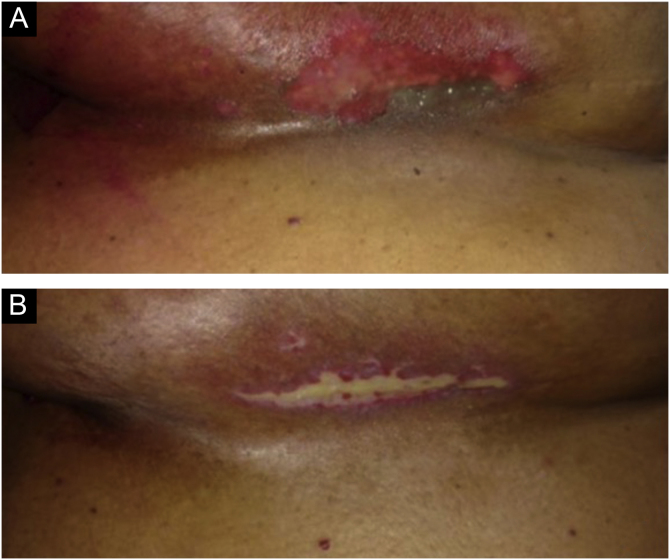
Images of the inframammary fold after 28 radiotherapy sessions with a total dose of 5654 cGy. (A) Grade II radiodermatitis, characterized by localized exulceration (moist desquamation). (B) Fourteen days after the end of radiotherapy shows grade IV radiodermatitis, characterized by ulceration.

Some degree of radiodermatitis occurred in 111/113 patients (98.2%). Regarding the severity, 95/113 patients (85.6%) developed grade I radiodermatitis, 14/113 (12.6%) grade II, 1/113 (0.9%) grade III, and 1/113 (0.9%) %) grade IV. It is noteworthy that among the patients who received booster radiotherapy, 54/68 (79.4%) developed radiodermatitis during the standard radiotherapy and maintained grade I, 12/68 (17.6%) developed grade II, and only six started the reaction after the booster treatment, with 1/68 (1.5%) developing grade III and 1/68 (1.5%) grade IV and both started the reaction after the booster. Figs. 1 to 3 represent patients who developed different degrees of radiodermatitis in the breast.

It is also important to highlight the characteristics of the two patients who did not develop any degree of radiodermatitis. One was aged 77 years, BMI = 25.9; received nine sessions, with an average dose of 2400 cGy, and did not continue the treatment due to her death. The other patient, 46 years old, BMI of 28.5, received only 10 sessions as prescribed by the radiotherapist for palliative treatment, also with an average dose of 2400 cGy.

### Secondary outcomes

#### Factors associated with radiodermatitis with higher severity degrees

All variables depicted in Table 1 were analyzed regarding their association with radiodermatitis of higher severity degrees (II, III and IV). The variables in which the p-value in the univariate analysis was < 0.1 were included in the logistic regression; therefore, the following parameters were included: body mass index, diabetes mellitus, dyslipidemia, use of oral hypoglycemic agents, analgesics and statins, variation in radiation dose, radiation depth, irradiated volume, and previous chemotherapy regimens. This analysis showed that for each unit of Body Mass Index (BMI) increase, the chance of occurrence of radiodermatitis grades II to IV increased by 14% (OR = 1.14; 95% CI 1.04‒1.26; p = 0.004) and that the use of statins concomitantly with radiotherapy increased the risk by four-fold (OR = 4.27; 95% CI 1.11‒16.42; p = 0.035).

The dose, fractionation, and energy received in the booster treatment for surgical scars were also evaluated. In the statistical analysis, these variables did not appear to be independent factors of radiodermatitis severity (p > 0.1).

#### Time of radiodermatitis onset

The mean time for the appearance of radiodermatitis was 17 ± 5 (range 5 to 27) days. In all cases, radiodermatitis manifested before the completion of standard radiotherapy and starting booster radiotherapy. It is noteworthy that 12/113 (10.6%) patients received silicone prostheses and all developed grade I radiodermatitis, with a mean time for onset of 18.4 ± 5.7 (variation of the time of onset of the reaction between 8 and 26) days.

**Figure 4:**
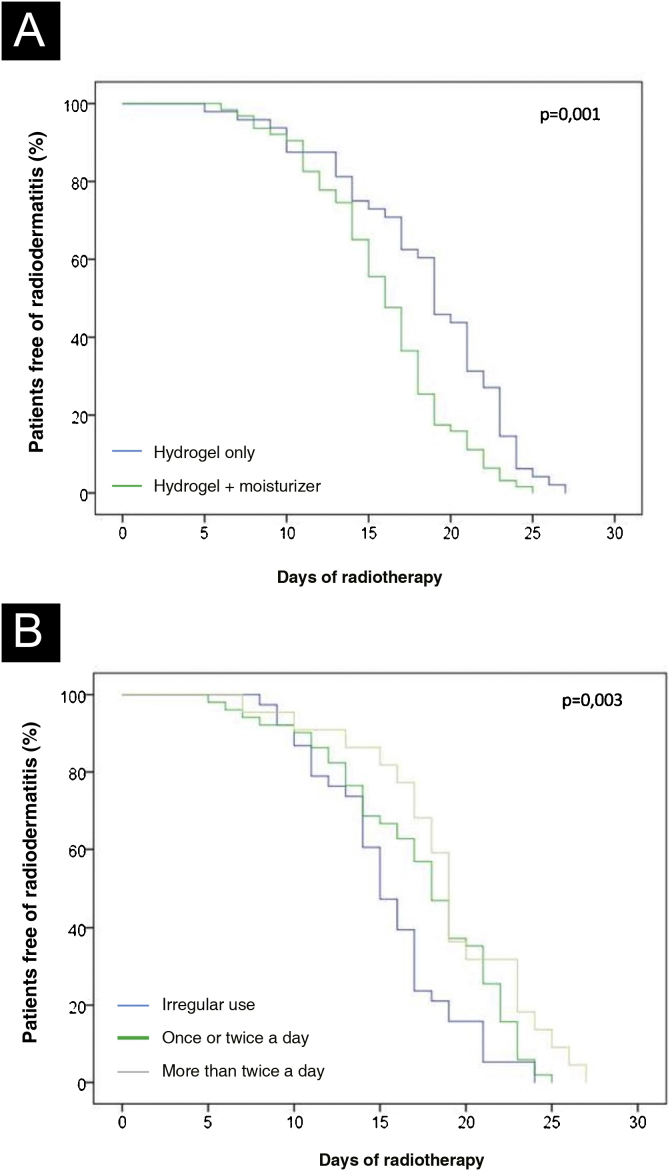
Kaplan-Meier curves. (A) Time of occurrence of radiodermatitis according to skin hydration regimen. (B) Time of occurrence of radiodermatitis according to the frequency of hydrogel use.

All variables shown in Table 1 were analyzed regarding their association with the time of onset of radiodermatitis. Multivariate analysis, performed using the Cox regression model, showed that the exclusive use of hydrogel for skin hydration was an independent factor in delaying radiodermatitis onset when compared to patients using hydrogel associated with moisturizing creams (p = 0.004), as shown in Table 2. The median time for the appearance of skin lesions in patients using hydrogel alone was 19.0 (range 14.5‒23.0) days, while for patients using hydrogel and moisturizing creams was 16.0 (range 13.0 to 19.0) days (p = 0.003). Fig. 4A shows the curve of patients free of radiodermatitis according to the skin hydration regimen used.

**Table 2:** Multivariate analysis of predictive factors for time to radiodermatitis onset in women with breast cancer treated by photon radiation therapy.

	Hazard Ratio (95% CI)	p-value
Vitamin supplementation	0.68 (0.40 – 1.14)	0.14
Previous chemotherapy		
DCT regimen (reference)^a^	‒	‒
Other regimens^b^	1.50 (0.89 – 2.52)	0.12
Not performed	1.03 (0.60 – 1.76)	0.93
Hydrogel use *vs.* hydrogel with moisturizer	0.55 (0.36 – 0. 82)	0.004

95% CI, 95% Confidence interval.

The parameters shown in this table, used for the Cox proportional hazards model, were those that showed a p-value < 0.1 in the univariate analysis.

a DCT regimen– Doxorubicin, Cyclophosphamide and Taxane.

b Other regimens – Cyclophosphamide plus taxane, Doxorubicin plus cyclophosphamide, Doxorubicin, cyclophosphamide and carboplatin, Doxorubicin, cyclophosphamide and fluorouracil, Taxane plus carboplatin.

Considering that the exclusive use of hydrogel delayed the onset of radiodermatitis by a few days, a more detailed analysis of this group of patients was carried out, which was divided according to the frequency of exclusive use of the hydrogel: irregular (less than once a day), once or twice a day and more than twice a day. The median time for the appearance of radiodermatitis in the last two groups that used the hydrogel more frequently was greater than in those with irregular use (> 2 times/day: 19.0 [17.0‒23.0] days; 1‒ 2 times/day: 18.0 [14.0‒22.0] days; irregular use: 15.0 [13.0–17.0] days, p = 0.009). Fig. 4B shows the curve of patients free of radiodermatitis according to the frequency of hydrogel used for skin hydration during the radiotherapy period.

## Discussion

In the present study, patients with breast cancer underwent two-dimensional radiotherapy with a linear accelerator and almost all of them (98.2%) had radiodermatitis in the breast; this reaction usually started after a few sessions. The only two patients who did not have radiodermatitis were those who received around 10 sessions, therefore with a lower accumulated dose.

These findings are very similar to those found by other studies with a high incidence rate of this toxicity.^3^, ^17^ Yee et al. conducted a systematic review including 96 randomized clinical trials with breast cancer patients who underwent external beam radiotherapy and found a variable incidence between studies from 26.3% to 92.2%. They concluded that modes of radiotherapy administration such as Intensity-Modulated Radiotherapy (IMRT) and hypofractionation have been shown to decrease skin toxicity compared to conventional treatments.^3^

The study by Lee et al. observed an incidence very similar to that of the present study, with a 97.3% incidence of radiodermatitis in 111 women with breast cancer in South Korea; however, they did not report the radiotherapy technique used.^18^ A high rate of cutaneous toxicity was also observed in a Brazilian study, carried out in a hospital in São Paulo with 86 women, in which 100% of the patients developed this reaction after radiotherapy with a linear accelerator, with a total dose of 5040 cGy (180 cGy dose/day).^19^ This high incidence corroborates the importance of studying radiodermatitis, especially with regard to measures to prevent or delay its onset.

A striking finding in this study was the impact of BMI on the incidence of higher degrees of radiodermatitis. For each additional BMI unit, there was a 14% increase in the risk of grades II to IV skin reactions. Other studies have also identified an association between BMI and an increased risk of radiodermatitis, finding an association of BMI with the development of severe breast skin reactions.^13^ In another study, Twardella et al. also demonstrated an association between BMI and an increased risk of acute skin toxicity.^12^ These authors also identified a correlation between large breasts and higher BMI. This finding could justify BMI as an independent risk factor for more severe radiodermatitis. In a systematic review, Mukesh et al. found that greater breast volume was related to the risk of radiodermatitis.^14^ Also, it can be said that women with higher BMIs are likely to have larger breasts and, consequently, a greater risk of skin toxicity resulting from radiation. The justification for these findings in larger breasts is due to the presence of extra tissue, which interferes with radiation absorption because of non-homogeneous dose distribution and greater separation field. There is also a greater drop of the breast into the mammary fold causing greater toxicities in this region; this occurs because the redundant tissue in the inframammary region serves as a zone of accumulation of the photon beam dose, which is then incident on the tissue opposite to it.^12^

Another finding in this study was the association between the continued use of statins and the development of more severe radiodermatitis. This class of drugs increased the risk of patients experiencing grades II to IV skin toxicity by four-fold. There is limited data in the literature confirming this association, which has been described as radiation recall dermatitis (RRD). In this phenomenon, the skin lesion appears after the end of radiotherapy. It is best described for chemotherapy after radiotherapy. However, two cases of RRD with the use of statins have been reported in the literature. Abadir & Liebmann described the case of a patient with bladder cancer who received local radiotherapy and did not develop skin lesions during radiotherapy.^20^ However, one year after the end of radiotherapy and beginning of the use of simvastatin for hypercholesterolemia, the patient developed a lesion at the site of the previous irradiation. A similar report was made by Taunk et al., who described the case of a patient with breast cancer undergoing radiotherapy who developed radiodermatitis during treatment.^21^ After five years, the patient started using rosuvastatin and amlodipine to treat hypercholesterolemia and systemic arterial hypertension. After two weeks, she developed a skin lesion on the site where she had previously received irradiation consistent with radiodermatitis. The question that arises is whether statins have any deleterious effects on the skin, especially when associated with radiotherapy. This class of drugs inhibits the enzyme 3-hydroxy-3-methylglutaryl coenzyme A (HMG CoA) reductase, causing a significant reduction in serum cholesterol levels, especially those of low-density lipoprotein cholesterol. With this effect on HMG CoA reductase, statins inhibit the mevalonate pathway and consequently reduce the production of non-steroidal isoprenoid compounds, which results in pleiotropic cell effects including cell proliferation, differentiation, and myelination.^22^ Reports of skin lesions induced by statin use have been presented.^23^, ^24^, ^25^, ^26^ Considering that radiation chemically damages the skin, perhaps the use of statins can potentiate the damage in the skin regeneration process. Another factor that should be mentioned, although not evaluated in this study, is drug interactions with statins, which may increase the risk of side effects from these drugs.

Another important finding of this study was the use of hydrogel as a protective factor against radiodermatitis. Although hydrogel use did not prevent the occurrence of radiodermatitis, as only two participants did not have skin toxicity, it delayed its onset when compared to patients who used hydrogel combined with moisturizing creams. In the authors’ service, all patients undergoing radiotherapy are instructed to moisturize their skin three times a day, either with moisturizer or hydrogel. The recommendation of which product should be used for hydration depends on product accessibility for each patient. As moisturizing creams were used as an alternative to hydrogel, mainly due to the higher cost of hydrogel, patients who used both products mostly belonged to the group who used hydrogel irregularly. There was no group of patients who used only moisturizing cream on a regular basis, making it impossible to draw more reliable conclusions about the best way to delay radiodermatitis. The present study design, as an observational one, is not the best to evaluate the effectiveness of interventions. To reduce confounding factors, the appropriate study design is a clinical trial, preferably randomized, with the formation of standardized and more homogeneous treatment groups.

However, the use of hydrogel as a protective factor against radiodermatitis has already been demonstrated. Censabella et al. conducted a study comparing patients divided into a group that used hydrogel throughout radiotherapy (preventive hydrogel group) and two groups of matched historical controls: one that applied dexpanthenol cream throughout therapy (dexpanthenol group) and one who applied dexpanthenol cream for 11‒14 sessions of radiotherapy followed by hydro active gel (curative hydrogel group). The patients in the preventive hydrogel group developed less radiodermatitis and later than patients in the dexpanthenol group and did not differ from the curative hydrogel group.^27^

Skin hydration is known to prevent skin toxicity from radiotherapy.^28^, ^29^ Skin capacity to retain water is related to the outer stratum corneum of the epidermis^30^ and it can be retained by hydration of corneocytes, the cells that make up the stratum corneum. The intracellular lipids in this layer form a transepidermal barrier that prevents water loss. The sebaceous glands also play an important role in water retention, mainly by producing glycerol. When the skin is irradiated, significant damage occurs, affecting the production of glycerol and intracellular lipids, allowing water loss and subsequent skin dryness, which contributes to the effects of radiation on the skin and the development of radiodermatitis.^6^

In addition to the adverse events inherent to radiotherapy, a Brazilian study showed that women who underwent radiotherapy for breast cancer had more frequent dermatological complaints, which was statistically significant (PR = 1.13; 95% CI 1.03 to 1.23; p = 0.011). These complaints were mainly related to radiodermatitis, such as erythema, hyperpigmentation, xerosis, and breast pruritus, demonstrating the impact of these dermatological changes in patients being treated for breast cancer.^31^

The main limitation of this study was its external validity. The factors identified here should be considered for services and populations similar to those in the present study. Radiotherapy in this sample was performed with planning based on flat images (two-dimensional images). Some services use a different radiotherapy technique with image-based volumetric planning (three-dimensional). Iwakawa et al. previously demonstrated that the risk of skin reactions in the breast was highly dependent on the institution where the patient was treated, due to different treatment techniques, lack of dose homogeneity, and the three-dimensional breast shape.^9^ For this reason, this study was carried out to identify the characteristics of radiodermatitis with this type of radiotherapy. Another limitation was not performing breast volumetry, since larger breasts were identified in another study as an independent risk factor for greater radiodermatitis severity.^32^ However, in the present study, the irradiated volume was analyzed, which is a variable related to breast volume, but it did not appear to be an independent risk factor for severity and time of onset of radiodermatitis in the multivariate analysis. Also, the patients were not classified according to Fitzpatrick phototypes, but according to the self-reported ethnicity, showing a predominance of white ethnicity (92.1%) and this was not a factor related to the severity and time of onset of radiodermatitis. The study by Twardella et al., which classified patients according to phototype, did not find this variable to be a risk factor.^12^

Finally, the findings of the present study are valid for radiotherapy with planning based on flat images, two-dimensional radiotherapy technique with a linear accelerator, and X-ray beams (photons), with a mean dose of 5,727 cGy.

## Conclusion

The present study contributes to a better understanding of radiodermatitis in women with breast cancer, as it shows its high incidence, the relationship of its severity with BMI and the use of statins, and the protective effect of hydrogel use. Future studies are required to increase knowledge about the factors associated with radiodermatitis, considering different populations and the diversity of treatments.

## Financial support

None declared.

## Authors' contributions

Loren Giagio Cavalcante: Design and planning of the study; drafting and editing of the manuscript, collection, analysis, and interpretation of data; literature review; critical review of the manuscript; approval of the final version of the manuscript.

Rejane Aparecida Rodrigues Domingues: Data collection; literature review; critical review of the manuscript; approval of the final version of the manuscript.

Batista de Oliveira Junior: Analysis and interpretation of data; critical review of the manuscript; approval of the final version of the manuscript.

Marco Antônio Rodrigues Fernandes: Analysis and interpretation of data; critical review of the manuscript; approval of the final version of the manuscript.

Eduardo Carvalho Pessoa: Analysis and interpretation of data; critical review of the manuscript; approval of the final version of the manuscript

Luciana Patrícia Fernandes Abbade: Effective participation in research orientation; analysis and interpretation of data; statistical analysis; drafting and editing of the manuscript; literature review; critical review of the manuscript; approval of the final version of the manuscript.

## Conflicts of interest

None declared.
